# Characteristics of Exposure to Chloromethylisothiazolinone (CMIT) and Methylisothiazolinone (MIT) among Humidifier Disinfectant-Associated Lung Injury (HDLI) Patients in South Korea

**DOI:** 10.3390/molecules25225284

**Published:** 2020-11-12

**Authors:** Dong-Uk Park, Seon-Kyung Park, Jiwon Kim, Jihoon Park, Seung-Hun Ryu, Ju-Hyun Park, So-Yeon Lee, Han Bin Oh, Sungkyoon Kim, Kyung Ehi Zoh, Soyoung Park, Jung-Hwan Kwon

**Affiliations:** 1 Department of Environmental Health, Korea National Open University, Seoul 03087, Korea; jiwonk1012@mail.knou.ac.kr; 2 Division of Environmental Science and Ecological Engineering, Korea University, Seoul 02841, Korea; kniel@hanmail.net; 3 Accident Response Division, National Institute of Chemical Safety, Ministry of Environment, Daejeon 34111, Korea; ichkann@korea.kr; 4 Humidifier Disinfectant Health Center, National Institute of Environmental Research, Incheon 22689, Korea; rsh1983@naver.com; 5 Department of Statistics, Dongguk University, Seoul 04620, Korea; juhyunp@gmail.com; 6 Department of Pediatrics, Childhood Asthma Atopy Center, Humidifier Disinfectant Health Center, Asan Medical Center, University of Ulsan College of Medicine, Seoul 05505, Korea; imipenem@hanmail.net; 7 Department of Chemistry, Sogang University, Seoul 04107, Korea; hanbinoh@sogang.ac.kr; 8 Department of Environmental Health Sciences, Institute of Health and Environment, Graduate School of Public Health, Seoul National University, Seoul 08826, Korea; ddram2@snu.ac.kr (S.K.); kezoh@snu.ac.kr (K.E.Z.); 9 Department of Occupational and Environmental Medicine, Kangbuk Samsung Hospital, School of Medicine, Sungkyunkwan University, Seoul 03181, Korea; syoem.park@samsung.com

**Keywords:** humidifier disinfectant-associated lung injury (HDLI), humidifier disinfectant (HD), chloromethylisothiazolinone (CMIT), methylisothiazolinone (MIT), inhalation exposure, patient

## Abstract

This study aimed to quantify both chloromethylisothiazolinone (CMIT) and methylisothiazolinone (MIT) dissolved in different product brands and to characterize the exposure to these chemicals among humidifier disinfectant-associated lung injury (HDLI) patients. Both CMIT and MIT dissolved in different humidifier disinfectant (HD) products were quantified using gas chromatography–mass spectrometry. The inhalation level of CMIT and MIT was estimated based on HD-associated factors as reported by HDLI patients. A total of eleven HD products marketed until the end of 2011 were found to contain CMIT and/or MIT. The level of combined CMIT and/or MIT dissolved in these HD products ranged from 12 to 353 ppm. The level varied among HD products and the year of manufacture. The average inhalation levels were estimated to be 7.5, 4.1, and 3.2 μg/m^3^ for the definite, probable, and possible groups, respectively. If probable and possible groups were collapsed together, the inhalation level of the collapsed group was significantly different from that of the definite group (*p* < 0.001). All HDLI patients responded as having used HD not only while sleeping, but also as having a humidifier treated with HD within close proximity every day in insufficiently ventilated spaces. These HD use characteristics of patients may be directly/indirectly linked to the HDLI development.

## 1. Introduction

A number of studies have demonstrated that chemicals added to a household humidifier as a disinfectant to suppress microbial growth have caused fatal lung injury, including interstitial pneumonitis and widespread lung fibrosis in children, pregnant women, and even adults. These specific lung injuries have been collectively named humidifier disinfectant-associated lung injury (HDLI) [1,2,3]. From May 2013 to date, the Korean government has operated the Humidifier-associated Lung Injury Investigation and Decision Committee (HLIIDC) to evaluate whether the injuries of people who register with the committee are clinically associated with humidifier disinfectant (HD) use [4]. The HLIIDC found that the chemicals causing HDLI include polyhexamethylene guanidine phosphate (PHMG, CAS No. 89697-78-9), oligo(2-(2-ethoxy) ethoxyethyl guanidinium (PGH, CAS No. 374572-91-5), or a mixture of 5-chloro-2-methyl-4-isothiazolin-3-one (CMIT, CAS No. 26172-55-4), and 2-methyl-4-isothiazolin-3-one (MIT, CAS No. 2682-20-4) [4].

A total of 453 (38%) among the 1199 people who registered with the HLIIDC program from 2013 to 2017 were clinically evaluated as to whether their lung injury was associated with the use of HD. The HDLI patients who responded as having used only HD containing PHMG accounted for 52% (*n* = 234), followed by only products containing PGH (*n* = 27.6%), and only a mixture of CMIT and MIT (*n* = 26.6%). The remaining HDLI patients responded indicating the use of several products of HD over time, resulting in mixed exposure to these disinfectants [5]. Isothiazolone and its derivatives have been widely used as preservatives to control microbial growth in a large number of industrial and household products [6,7]. Basic information on the physicochemical properties of CMIT and MIT, including usages related to this study, is indicated in Table 1 [8,9,10]. A mixture of 3:1 CMIT and MIT under the brand Kathon CG has been extensively used in water-based personal products and cosmetic applications as a preservative, causing many known cases of eczema, contact allergy and dermatitis [11,12], and other skin allergic reactions [11,12,13,14,15]. The use of a CMIT/MIT mixture is currently banned in Korea for cosmetics, except for rinse-off products with a permitted concentration of up to 0.0015% [16]. These chemicals were first used as disinfectants in household humidifiers in South Korea. To date, lung injury patients have been reported among people who used HD. However, the characteristics of exposure to CMIT and MIT among HDLI patients, which could be used for assessment of the role of CMIT and MIT in the development of the disease, have been poorly defined. This study aimed to quantify both CMIT and MIT levels dissolved in HD products and to characterize the inhalation exposure level to these chemicals of HDLI patients (including estimated inhalation level).

## 2. Results

**Figure 1:**
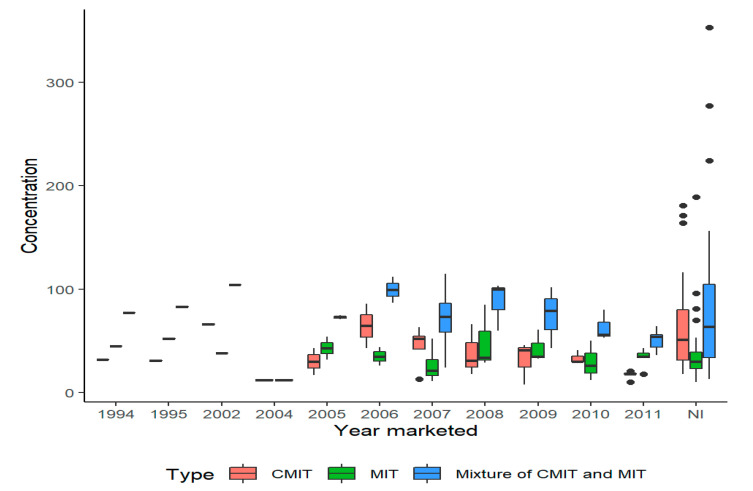
The levels of CMIT and MIT contained in the HD products by year marketed (CMIT, 5-chloro-2methylisothiazol-3(2*H*)-one; MIT, 2-Methylisothiazol-3(2*H*)-one).

**Figure 2:**
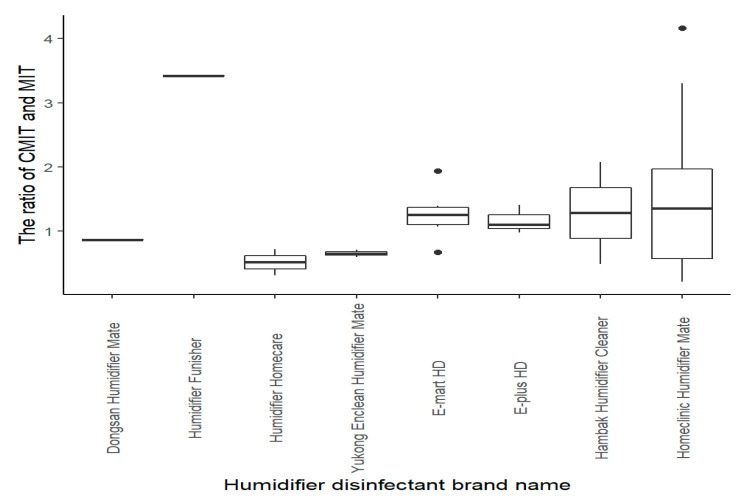
The distribution of the ratio of CMIT and MIT dissolved in HD by HD product (CMIT, 5-chloro-2methylisothiazol-3(2*H*)-one; MIT, 2-Methylisothiazol-3(2*H*)-one).

A total of 11 HD products were found to contain CMIT and/or MIT. From 1994 until the end of 2011, these HD products were widely marketed. The HD product named as Home Clinic Humidifier Mate showed the highest sales volume (Table 2). The level of combined CMIT and MIT in the 11 products was quantified ranging from 12.0 to 353.3 mg/L (*n* = 52), which varied according to the HD products and the year manufactured (Table 3 and Figure 1). Estimated inhalation levels of combined CMIT and MIT ranged from 0.3 to 8.1 μg/m^3^ among the HDLI patients. The ratio of CMIT and MIT ranged from 0.21 to 4.17, with an average of 1.43 (*n* = 44), and varied among HD products (Figure 2). The average inhalation levels were estimated to be 7.5 μg/m^3^, 4.1 μg/m^3^, and 3.2 μg/m^3^ for the definite, probable, and possible groups, respectively. A significant difference in estimated inhalation levels of HDLI patient groups (definite association of lung injury with HD use = 2, probable = 7, and possible = 17) was not found. If probable and possible groups were collapsed together, the inhalation level of the collapsed group was significantly different from that of the definite group (*p* < 0.001). Most of the HDLI patients (*n* = 20) were found to be younger than seven years of age. All HDLI patients responded as having used HD not only while sleeping, but also as having kept a humidifier within two meters every day for several months, except for one patient who used a humidifier for one month (Table 4).

## 3. Discussion

We characterized the HD inhalation exposure of 26 HDLI patients who responded as having used only HD products containing CMIT and MIT, focusing on an estimation of inhalation exposure. HDLI patients have been clinically confirmed in the consecutive HLIIDC programs [1], in which clinicians were blinded regarding information on types of HD and HD product. There has been controversy in Korea over whether CMIT and MIT posed a risk of lung injury due to the small number of HDLI patients using them, especially “definite” (*n* = 2), “probable” (*n* = 7) and “possible” HD association cases (*n* = 17), relative to those involving PHMG (“definite” = 72, “probable” = 53, “possible” = 67) [5]. It is difficult to find a causal association through epidemiological study or animal testing in rare diseases with a small number of cases. There has been no study to examine how many people used only HD products containing CMIT and MIT and developed HDLI. The number of HDLI cases related to HD containing only a mixture of CMIT and MIT (*n* = 26) was obtained from only people who registered with the HLIIDC program before the end of 2017 (*n* = 1199). To date, no case–control epidemiologic studies have been conducted to examine the causal relationship between risk of HDLI and the use of HD including CMIT and MIT. Animal tests of inhalation exposure conducted in South Korea have not found symptoms related with lung injury [17,18], although a recent animal test using intra-tracheal administration showed a similar interstitial lung injury as that found in HDLI patients [19]. Both HD exposure characteristics [4,5] and clinical manifestations of HDLI patients who used HD containing PHMG have been reported elsewhere [1,2,3,20,21] along with a significant association and causal relationship in animal testing. Two HDLI patient sisters who used only a HD brand containing a mixture of CMIT and MIT (Table 4) were reported to show the typical rapid progression of respiratory difficulty, starting from a mild cough or tachypnea [22] and resulting in peripheral airway dysfunction, similar to HDLI caused by exposure to PHMG or PGH. Another case report demonstrating HDLI pathology proved that CMIT/MIT can also cause pathologically similar lung injury due to PHMG or PGH [22]. HD exposure characteristics of HDLI patients who used only HD containing a mixture of CMIT and MIT are discussed here to link with the risk of HDLI, focusing on the estimated inhalation level.

Firstly, the combined level of CMIT and MIT dissolved in HD products (range: 11.8 to 353.3 ppm, mean = 79.5 ppm) varies according to the HD product and year manufactured (even within the same product type). The levels of CMIT and MIT found in three samples from two HD products that had never been opened, with no possibility of loss or contamination (CMIT 32 ppm and MIT 45 ppm for the Yukong Enclean Humidifier Mate and CMIT 18.7 ppm, 17.7 ppm and MIT 34.9 ppm, 18.7 ppm for the Home Clinic Humidifier Mate, respectively) (Table 3) were found to be far lower than the concentrations calculated below according to the recommended procedures for manufacturing HD [23]. SKYBIO FG, a 1.5% solution of a 3:1 blend of CMIT and MIT, was diluted to manufacture the various HD products. This concentration (1.5% = 15,000 ppm) of CMIT and MIT from SKYBIO FG diluted for use in the HD products is approximately 1–2% for CMIT and 0.2–0.6% for MIT, resulting in approximate concentrations of 112.5–225 ppm (1.5% = 15,000 ppm × 0.75% × 0.01–0.02%) for CMIT and 7.5–22.5 ppm for MIT (1.5% = 15,000 ppm × 0.25% × 0.002–0.006%), based on the ratio of 3:1 CMIT to MIT. Magnesium nitrate (20–25%), magnesium chloride (0.2–1.0%) and water (70–75%) were also included in the HD products. Based on the physio-chemical properties of CMIT and MIT (Table 1), we cannot exclude the possibility of evaporation from the solution during storage. CMIT and MIT are electrophilic chemicals that are usually stable at pH ≤ 7 [24]. Slow reactions with the surfaces of the plastic container over time and potential evaporation would explain why the measured concentrations in the collected samples stored for a long time are much lower than what would be estimated assuming a typical manufacturing processes. In addition, the ratio of CMIT and MIT, as quantified in this study, did not follow the original ratio of 3:1, implying that CMIT was lost much easier than MIT was (Figure 2). The concentrations quantified from the fresh packages that have never been opened were either lower or higher than from the used samples (Table 3).

Secondly, the large variations and inconsistent levels of CMIT and MIT among types of HD (Table 3) the and year manufactured (Figure 1) indicated that the intended levels in the HD products described above were not planned or controlled in the final stages by a quality assurance program. Several product samples even showed no indication of the year of manufacture or lot number on the containers (indicated as “no information” in Table 3). This result demonstrates that all companies neglected to maintain a specific level of CMIT and MIT. Most companies were found to outsource the manufacture of their HD to an external contractor. No documentation that SKYBIO FG was legally allowed in the manufacture of HD has been found. The inhalation toxicity of both SKYBIO FG and the HD products has not been studied. A mixture of CMIT and MIT was widely used in HD products from 1994 through to the end of 2011. The use of this mixture has already been banned in cosmetic products due to consistent cases of health complaints but was permitted for rinse-off products [25,26]. The levels of a mixture of CMIT and MIT dissolved in several HD products (Table 3) are far higher than the maximum authorized concentration (15 ppm) in rinse-off cosmetic products in Korea, the EU, and the US [25,26]. In the United States, it is currently used in concentrations of up to 8 ppm in other cosmetics.

Thirdly, our estimated airborne levels of the mixture of CMIT and MIT to which HDLI patients were exposed (Table 4) are similar to the results (CMIT: 0.95–4.37 μg/m^3^, MIT: 0.30–1.75 μg/m^3^) reported by Park et al. (2020), who measured airborne CMIT and MIT using two serial impingers in a room-sized stainless steel chamber where HD containing the intended level was used [27]. This partly supports the validity of the assumptions for exposure assessment in this study, although there might be variations in exposure parameters among patients and potential losses of CMIT and MIT other than through ventilation. Seven of nine HDLI patients were found to use concentrations below the 10 mL recommended by the product (Table 4).

Fourthly, we assumed that a mixture of CMIT and MIT was dispersed from the HD into the air and inhaled to the respiratory tracts of the HDLI patients without any loss other than through ventilation. This assumption was based on several HD use characteristics; the use of HD in a small semi-enclosed room with little or no ventilation within two meters of the humidifier; intensive HD use every day during the dry period from late fall through winter or early spring; and HD use while sleeping (Table 4). It is very difficult to examine what level of CMIT and/or MIT can cause HDLI, especially in children under seven years of age. No airborne threshold level for CMIT and/or MIT has been recommended to prevent the respiratory health effects. No study has reported that CMIT and/or MIT causes the lung injuries developed by HDLI patients, despite its widespread use as a disinfectant for cosmetic, household and industrial products. The major exposure route of CMIT and/or MIT from most consumer products would be through the skin, resulting in little inhalation exposure alongside considerable skin exposure. To the best of our knowledge, only one case of respiratory disease related to CMIT or MIT has previously been reported, a chemical operator aged 53 in an isothiazolinone manufacturing plant who developed asthma five months after starting work [28].

Finally, no other significant individual factors causing fatal lung injury outside of the use of HD, including among the children HDLI patients, have been found in this study. No environmental or other factors with the potential to be genetically inherited have been found to relate to fatal lung injury. We could support this statement by checking whether parents of the patients had experienced fatal lung injury. For children, lung injury that cannot be explained by infection or environmental exposure is a characteristic finding of lung imaging, and a decrease in lung function was observed. Intensive and consecutive daily use of HD with a humidifier within two meters could be a major factor causing the development of fatal lung injury, directly or indirectly. Most HDLI patients continued to use HD every day causing a limitation in the biological ability to compensate for reversible or irreversible respiratory health problems, including lung injury [29]. Preschool children, in particular infants were exposed to HDs intensively during early infancy, a time when their lung development is incomplete. We reached the conclusion that there is no individual causation of fatal HDLI outside the use of HD containing a mixture of CMIT and MIT. We assumed that several HD exposure characteristics including inhalation exposure level of CMIT and MIT, HD use duration, HD use while sleeping, and distance from humidifier would likely contribute to the understanding of the risk of health problems, including HDLI. The upper respiratory tract and skin can be the first targets of CMIT and MIT. Further study is necessary to demonstrate how these chemicals with good water-solubility can be deposited in and damage the lower part of the respiratory tract, as in the case of HDLI.

This study has certain limitations. The number of samples by HD product and year manufactured were severely unbalanced because of the considerable differences in sales volumes and the manner of collection. Our HD samples (*n* = 53) were entirely collected from registered people who kept residual HD and are minute compared to the number and volume marketed, making it difficult to represent the levels dissolved in all HD products containing CMIT and MIT. The loss of electrophilic CMIT and MIT during a certain period of storage is another important limitation for estimating the level of CMIT and MIT in marketed HD products. The stability of CMIT and MIT in plastic containers during storage has never been studied. However, several other HD exposure or use variables employed in this study were potentially appropriate for evaluating the qualitative association with the risk of HDLI, even though there is a limitation in examining quantitative association due to the small number of non-randomized HDLI patients (*n* = 26). The possibility of recall bias in the HD use-related information could be low considering the daily HD use over less than one year, as well as the development of fatal lung injury in early childhood.

## 4. Materials and Methods

### 4.1. Collection of Humidifier Disinfectant (HD) Samples

All HD samples were collected during the environment investigation visit with people who registered with the HLIIDC program. Samples were stored in polyethylene bottles, transported by ice box, and stored in a refrigerator until their analysis. All HD products that were suspected to contain CMIT and/or MIT were quantified. The methods applied to evaluate the use characteristics of HDs based on personal interviews and home investigations have been described elsewhere [30,31]. Trained environmental health scientists visited registered patients’ homes and conducted personal interviews and home investigations with the patients and their family members in order to complete detailed questionnaires or checklists related to HD use. All exposure-related variables used in this study were selected from the survey results of the HLIIDC program.

### 4.2. Quantification of CMIT and MIT

CMIT (99%) and MIT (98%) were purchased from Dr. Ehrenstorfer GmbH (Augsburg, Germany) and Sigma-Aldrich (St Louis, MO, USA), respectively. Anhydrous sodium sulfate (99%) and alumina oxide (98%) used for matrix solid-phase dispersion (MSPD) were purchased from Sigma-Aldrich. Sodium sulfate was baked at 400 °C for six hours, and alumina oxide was activated at 190 °C for 12 h prior to use. The MSPD method was used to extract CMIT and MIT from liquid HD products. Detailed experimental procedure is described in a previous study [24]. A mixture of 2.0 g of baked sodium sulfate and 0.02 mL of liquid sample was mixed by gentle hand agitation. After the liquid samples were absorbed by the sorbent, 2.0 g of alumina oxide was added and the mixture was transferred into an extraction cartridge. Ten milliliters of acetone was flowed through the cartridge to elute CMIT and MIT, and the extract was analyzed using gas chromatography–mass spectrometry (GC-MS). The amounts of CMIT and MIT were quantified using a GC-MS system (Agilent 7890A GC and 5975C mass spectrometry detector (MSD); Agilent Technologies, Santa Clara, CA, USA) after the MSPD elution. A 1.0 μL extract was injected at 250 °C in splitless mode and the analytes were separated on an Agilent DB-5 MS capillary column (30 m × 0.25 mm; film thickness of 0.25 µm) using helium as the carrier gas at a constant flow rate of 1 mL/min^−1^. The GC oven temperature was initially 60 °C (held for 1 min), ramped up to 150 °C (held for 4 min) at 10 °C min^−1^ and then increased to 280 °C (held for 2 min) at 60 °C min^−1^. The MSD was operated in electron ionization (EI) mode (70 eV) and CMIT and MIT were determined at the selected mass-to-charge ratios (*m*/*z*) of 149 and 85 for CMIT and 115 and 87 for MIT.

The method detection limits (MDLs) for CMIT and MIT were determined using the error distribution method [32]. CMIT and MIT were spiked into deionized water at concentrations greater than two to three times the estimated MDLs and analyzed seven times following the same procedure used for samples. The MDL values were estimated by multiplying the standard deviation of the measurements by the corresponding *t* value for the sample size (3.707). MDLs were determined three times, and ranged from 4.3 to 10.9 mg/L and from 1.8 to 9.3 mg/L for CMIT and MIT, respectively. The samples with concentrations below the upper bound of the method detection limits (CMIT = 10.9 ppm, MIT = 9.3 ppm) were excluded. Owing to the diversity of the sample matrices of the HD samples, extraction recoveries were also determined for individual products using the fortification method [8]. Recoveries were generally higher for CMIT than MIT, and ranged between 47 and 187% for CMIT and between 12 and 111% for MIT.

### 4.3. Estimation of Inhalation Exposure Level

The inhalation exposure levels of 26 HDLI patents were estimated here based on their HD use characteristics. The method to estimate airborne HD concentration (μg/m^3^) was described elsewhere in detail [30] and is described briefly below. Airborne CMIT and MIT were estimated based on the dissolved concentration in the HD products (Table 2) and according to the HD use characteristics that the 26 HDLI patients reported.

- Amount of the mixture of CMIT and MIT dispersed into the air per hour (G, μg/h) = (C_1_ × V_1_)/T. This (G) was calculated by multiplying the bulk level (C_1_, μg/mL) and the total HD volume used per day (V_1_, mL) and then dividing by average HD use hours (T, h). The concentrations of CMIT and MIT dissolved in HD products (C_1_) were matched with the products HDLI patients responded as “use” (Table 2).
- Estimated air flow rate (Q, m^3^/h) = size of room in which HD was used (m^3^, V_2_) × air change per hour (ACH) in the room. The size of the room in which HD was used was measured during the home visit. ACH was assumed to be 0.5 based on the level of ventilation in a semi-confined room because the HDs were mostly used from late fall through winter to early spring. HDLI patients responded as having used HD containing a mixture of CMIT and MIT during the winter season while sleeping.
- Estimated inhalation concentration (C_2_, μg/m^3^) = G/Q.

Lung injury in study subjects (*n* = 26) was clinically evaluated to have either a definite, probable, or possible association with HD use. The clinical confirmation of lung disease cases was performed by a committee composed of pediatric and adult pulmonologists, respiratory pulmonologists, radiologists, pathologists, and epidemiologists without information on the type of HD but based on a combination of clinical manifestations, natural disease courses, and radiological and pathological findings in subjects for whom lung specimens were available [2]. The clinical evaluation results were eventually matched with HD exposure characteristics, including type of HD product and HD. Descriptive analyses and *F*-tests for comparing average inhalation levels among HDLI patient groups were performed using STATA 12.0 (STATA Corp, College Station, TX, USA) and R software (ver. 3.6.1, The R Foundation for Statistical Computing, Vienna, Austria).

## 5. Conclusions

This study found that HDLI patients used a HD containing a mixture of CMIT and MIT in semi-enclosed rooms and were situated within close proximity to the humidifier every night. They were consistently exposed to a mixture of CMIT and MIT ranging from at least 0.4 to 9.4 μg/m^3^ during late fall through the winter. These HD exposure characteristics could likely be linked to the development of HDLI, either directly or indirectly.

## Figures and Tables

**Table 1:** Basic information on the physicochemical properties of CMIT and MIT.

Property	CMIT	MIT
Chemical name (INCI)	Methylchloroisothiazolinone	Methylisothiazolinone
Preferred IUPAC name	5-chloro-2methylisothiazol-3(2*H*)-one	2-Methyl-1,2-thiazol-3(2*H*)-one
Other names	5-Chloro-2-methyl-4-isothiazolin-3-one Chloromethylisothiazolinone (CMI) Chloromethylisothiazolone (CMIT) Methylchloroisothiazolinone (MCI) Methylchloroisothiazolone (MCIT)	2-Methylisothiazol-3(2*H*)-one 2-Methyl-4-isothiazolin-3-one
CAS number	26172-55-4	2682-20-4
Chemical formula	C_4_H_4_CINOS	C_4_H_5_NOS
Molecular weight (g/mole)	149.59	115.16
Vapor pressure (Pa, 25 °C) ^†^	2.39	3.41
Vapor pressure (mmHg at 25 °C) ^†^	0.328	0.328
Melting point (°C)	52	50–51
Density (g/cm³)	1.51	1.29
Henry’s law constant (Pa m^3^/mol (20 °C, pH 5) ^1^	1.1 × 10^−5^	3.0 × 10^−4^
Formula	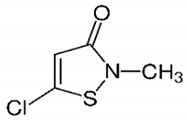	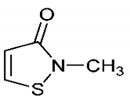
Usage	Preservatives with antibacterial and antifungal effects in combination with methylisothiazolinone for personal products and a wide range of industrial applications	

**Abbreviations:** INCI, International Nomenclature of Cosmetic Ingredients; IUPAC, International Union of Pure and Applied Chemistry; CAS, Chemical Abstract Service; EPA, United States Environmental Protection Agency; CMIT, 5-chloro-2methylisothiazol-3(2*H*)-one; MIT, 2-Methylisothiazol-3(2*H*)-one. ^1^ This value varies slightly within the literature. ^†^ Varying slightly among literatures.

**Table 2:** Humidifier disinfectant (HD) products containing CMIT and MIT.

Product Name	Raw Product Used	Volume (mL)	Sale Period	Number of Product Sales (mL/each)
Dongsan Humidifier Mate	NI	NI	NI	NI
Humidifier Furnisher	NI	1000	2007–2011	27,565
Humidifier Homecare	NI	NI	NI	NI
SK Humidifier Mate	SKYBIO-FG	230, 500, 1000	2001–2004	195,038
E-plus HD	NI	500, 1000	2006–2011	354,994
Yukong Enclean Humidifier Mate	SKYBIO-FG	230	1994–1996	353,601
HD Partner	NI	NI	NI	NI
Homekeeper	SKYBIO-FG	1000	2007–2009	11,028
Hambak HD	SKYBIO-FG	500, 1000	2007–2011	18,174
E-mart HD	SKYBIO-FG	500, 1000	2006–2011	354,994
Home Clinic Humidifier Mate	SKYBIO-FG	500, 1000	2002–2011	1,716,883

**Abbreviations:** CMIT, 5-chloro-2methylisothiazol-3(2*H*)-one; MIT, 2-Methylisothiazol-3(2*H*)-one; HD, humidifier disinfectant; NI, no information.

**Table 3:** The level of CMIT and MIT by year manufactured and humidifier product.

Product Name	Sales Period	N	CMIT (ppm, μg/mL)				MIT (ppm, μg/mL)				Mixture of CMIT/MIT (ppm, μg/mL)			
			Mean	SD	Min	Max	Mean	SD	Min	Max	Mean	SD	Min	Max
Dongsan Humidifier Mate	NI	2	164.0	-	164.0	164.0	135.0	76.4	81.0	189.0	217.0	192.3	81.0	353.0
Humidifier Furnisher	2010	1	41.0	-	41.0	41.0	12.0	-	12.0	12.0	53.0	-	53.0	53.0
Humidifier Homecare	NI	1	18.0	-	18.0	18.0	25.0	-	25.0	25.0	43.0	-	43.0	43.0
	2005	1	17.0	-	17.0	17.0	54.0	-	54.0	54.0	71.0	-	71.0	71.0
	subtotal	2	17.5	0.7	17.0	18.0	39.5	20.5	25.0	54.0	57.0	19.8	43.0	71.0
Yukong Enclean Humidifier Mate	1994 ^1^	1	32.0	-	32.0	32.0	45.0	-	45.0	45.0	77.0	-	77.0	77.0
	1995	1	31.0	-	31.0	31.0	52.0	-	52.0	52.0	83.0	-	83.0	83.0
	subtotal	2	31.5	0.7	31.0	32.0	48.5	4.9	45.0	52.0	80.0	4.2	77.0	83.0
E-mart HD	NI	1	30.0	-	30.0	30.0	23.0	-	23.0	23.0	53.0	-	53.0	53.0
	2007	1	63.0	-	63.0	63.0	52.0	-	52.0	52.0	115.0	-	115.0	115.0
	2008	2	48.5	24.7	31.0	66.0	31.5	3.5	29.0	34.0	80.0	28.3	60.0	100.0
	2009	2	43.5	3.5	41.0	46.0	47.0	19.8	33.0	61.0	90.5	16.3	79.0	102.0
	subtotal	6	46.2	15.5	30.0	66.0	38.7	14.6	23.0	61.0	84.8	24.9	53.0	115.0
E-plus HD	NI	2	46.5	12.0	38.0	55.0	38.5	16.3	27.0	50.0	85.0	28.3	65.0	105.0
	2006	1	43.0	-	43.0	43.0	44.0	-	44.0	44.0	87.0	-	87.0	87.0
	subtotal	3	45.3	8.7	38.0	55.0	40.3	11.9	27.0	50.0	85.7	20.0	65.0	105.0
Jupusarang Humidifier Partner	NI	1	-	-	-	-	31.0	-	31.0	31.0	31.0	-	31.0	31.0
Hambak Humidifier Cleaner	NI	2	19.0	-	19.0	19.0	32.0	9.9	25.0	39.0	41.5	23.3	25.0	58.0
	2007	1	52.0	-	52.0	52.0	25.0	-	25.0	25.0	77.0	-	77.0	77.0
	subtotal	3	35.5	23.3	19.0	52.0	29.7	8.1	25.0	39.0	53.3	26.3	25.0	77.0
Home Clinic Humidifier Mate	NI ^1^	17	74.5	52.5	19.0	181.0	33.1	22.4	10.0	96.0	90.0	75.1	13.0	277.0
	2002	1	66.0	-	66.0	66.0	38.0	-	38.0	38.0	104.0	-	104.0	104.0
	2004	1	-	-	-	-	12.0	-	12.0	12.0	12.0	-	12.0	12.0
	2005	1	43.0	-	43.0	43.0	32.0	-	32.0	32.0	75.0	-	75.0	75.0
	2006	1	86.0	-	86.0	86.0	26.0	-	26.0	26.0	112.0	-	112.0	112.0
	2007	2	32.5	27.6	13.0	52.0	14.5	4.9	11.0	18.0	47.0	32.5	24.0	70.0
	2008	1	18.0	-	18.0	18.0	85.0	-	85.0	85.0	103.0	-	103.0	103.0
	2009	1	8.0	-	8.0	8.0	35.0	-	35.0	35.0	43.0	-	43.0	43.0
	2010	2	30.0	0.0	30.0	30.0	38.0	17.0	26.0	50.0	68.0	17.0	56.0	80.0
	2011^2^	5	17.2	4.2	10.0	21.0	33.6	9.4	18.0	43.0	50.8	10.9	36.0	64.0
	subtotal	32	51.9	45.0	8.0	181.0	33.2	20.2	10.0	96.0	77.0	59.1	12.0	277.0
Total		52	49.7	40.7	8.0	181.0	38.3	28.4	10.0	189.0	80.4	61.9	12.0	353.0

**Abbreviations:** N, the number of products; CMIT, 5-chloro-2methylisothiazol-3(2*H*)-one; MIT, 2-Methylisothiazol-3(2*H*)-one; SD, standard deviation; HD, humidifier disinfectant; NI, no information. ^1^ Sample had never been used. ^2^ Includes two samples that had never been used (CMIT 18.7 ppm, 17.7 ppm; MIT 34.9 ppm, 18.7 ppm).

**Table 4:** Estimated airborne CMIT and MIT levels based on HD use characteristics of humidifier disinfectant-associated lung injury (HDLI) patients with clinically definite, probable, and possible association with use of HD (*n* = 26)**.**

Sex	Age at Diagnosis	Name of HD Product Used	C_1_ (ppm, μg/mL)	V_1_ (mL)	Average Hours Per Day	G (μg/day)	V_2_ (m^3^)	Q (m^3^/day)	C_2_ (μg/m^3^)	Use While Sleeping	Distance from Humidifier (m)	Total HD Use Duration (months) ^1^	Total Cumulative HD Use Hours ^1^	Association of Lung Injury with the Use of HD ^2^
F ^3^	<1	Only Home Clinic Humidifier Mate	80.4	3	4	60.3	16.1	8.1	7.5	Yes	1–2	3	336	Definite
F ^3^	<1		80.4	3	4	60.3	16.1	8.1	7.5	Yes	1–2	3	336	Definite
F	<1		80.4	20	20	80.4	32.3	16.1	5.0	Yes	1–2	2	1120	Probable
F	<1		80.4	10	13	61.8	36.1	18.1	3.4	Yes	≤2	5	1820	Probable
F	3		80.4	10	12	67.0	30.6	15.3	4.4	Yes	<0.5	4	384	Probable
M	3		80.4	4	10	32.2	33.1	16.6	1.9	Yes	≤2	6	720	Probable
M	4		80.4	10	12	67.0	16.5	8.3	8.1	Yes	1–2	17	5712	Probable
F	6		80.4	3	12	20.1	30.6	15.3	1.3	Yes	0.5–1	4	384	Probable
M ^4^	29		80.4	20	14	114.9	50.0	25.0	4.6	Yes	<0.5	11	4312	Probable
F	48		80.4	10	13	61.8	33.6	16.8	3.7	Yes	0.5–1	14.3	5205	Possible
F	32		80.4	40	20	160.8	48.0	24.0	6.7	Yes	0.5–1	1	560	Possible
F	24		80.4	20	13	123.7	36.2	18.1	6.8	Yes	1–2	5	1820	Possible
M	47		80.4	1.7	13	10.5	53.9	26.9	0.4	Yes	1–2	32	11,648	Possible
F	4	Only E-Mart HD	80.4	2.5	11	18.3	22.3	11.1	1.6	Yes	<0.5	3	924	Possible
F	1	Only Home Clinic Humidifier Mate	80.4	2.5	12	16.8	29.8	14.9	1.1	Yes	1–2	15	5040	Possible
M	6		80.4	13.4	10	107.7	80.5	40.3	2.7	Yes	≤2	10	2800	Possible
F	1		80.4	13.4	14	77.0	80.5	40.3	1.9	Yes	1–2	10	3920	Possible
F	<1		80.4	11.4	12	76.4	16.3	8.2	9.4	Yes	≤2	19	3648	Possible
M	4		80.4	10	10	80.4	34.0	17.0	4.7	Yes	≤2	23	6440	Possible
F	1		80.4	10	10	80.4	34.0	17.0	4.7	Yes	≤2	17	4760	Possible
F	3	Only Home Clinic Humidifier Mate 60%, E-Mart HD 40%	82.2	4.3	12	29.4	67.5	33.7	0.9	Yes	1–2	12	1728	Possible
M	2		82.2	4.3	12	29.4	67.5	33.7	0.9	Yes	≤2	18	2592	Possible
F	2	Only Home Clinic Humidifier Mate	69.7	10	16	43.6	33.7	16.9	2.6	Yes	0.5–1	18	8064	Possible
F	5		69.7	4.3	11	27.2	26.4	13.2	2.1	Yes	1–2	15	1980	Possible
F	2		69.7	3.6	15	16.7	29.8	14.9	1.1	Yes	<0.5	4	720	Possible
F	11		69.7	5	4	87.1	55.2	27.6	3.2	Yes	0.5–1	15	1680	Possible

**Abbreviations:** CMIT, 5-chloro-2methylisothiazol-3(2*H*)-one; MIT, 2-Methylisothiazol-3(2*H*)-one; HD, humidifier disinfectant; C_1_, contained concentration; V_1_, average total volume per day; G, used amount of CMIT and MIT per day; V_2_, room volume; Q, estimated air flow rate (V_2_ × 0.5 (air change per hour—ACH)); C_2_, estimated airborne concentrations of CMIT and MIT (G/Q); M, male; F, female. ^1^ Months and hours actually used, ^2^ HDLI patients with clinically definite, probable, and possible association with use of HD containing CMIT and MIT (*n* = 26), ^3^ twin sisters, ^4^ deceased.
